# Molecular and functional characterization of reversible‐sunitinib‐tolerance state in human renal cell carcinoma

**DOI:** 10.1111/jcmm.18329

**Published:** 2024-05-02

**Authors:** Angela Zaccagnino, Bozhena Vynnytska‐Myronovska, Michael Stöckle, Kerstin Junker

**Affiliations:** ^1^ Department of Urology and Pediatric Urology Saarland University Homburg Germany

**Keywords:** acquired drug‐tolerance, renal cell carcinoma, sunitinib, targeted therapy, tyrosine kinases

## Abstract

Therapy failure with the tyrosine kinase inhibitor (TKI) sunitinib remains a great challenge in metastatic renal cell carcinoma (mRCC). Growing evidence indicates that the tumour subpopulation can enter a transient, non‐mutagenic drug‐tolerant state to endure the treatment underlying the minimal residual disease and tumour relapse. Drug tolerance to sunitinib remains largely unexplored in RCC. Here, we show that sunitinib‐tolerant 786‐O/S and Caki‐2/S cells are induced by prolonged drug treatment showing reduced drug sensitivity, enhanced clonogenicity, and DNA synthesis. Sunitinib‐tolerance developed via dynamic processes, including (i) engagement of c‐MET and AXL pathways, (ii) alteration of stress‐induced p38 kinase and pro‐survival BCL‐2 signalling, (iii) extensive actin remodelling, which was correlated with activation of focal adhesion proteins. Remarkably, the acute drug response in both sensitive and sunitinib‐tolerant cell lines led to dramatic fine‐tuning of the actin‐cytoskeleton and boosted cellular migration and invasion, indicating that the drug‐response might depend on cell state transition rather than pre‐existing mutations. The drug‐tolerant state was transiently acquired, as the cells resumed initial drug sensitivity after >10 passages under drug withdrawal, reinforcing the concept of dynamic regulation and phenotypic heterogeneity. Our study described molecular events contributing to the reversible switch into sunitinib‐tolerance, providing possible novel therapeutic opportunities in RCC.

## 1 INTRODUCTION

Renal cell carcinoma (RCC) represents the seventh predominant type of neoplasm in developed countries, accounting for about 2% of all diagnosed cancers worldwide.^1^, ^2^ Clear cell carcinoma (ccRCC) embodies the most common histological subtype (65%–70%)^3^, ^4^ and is characterised by mutation of the *von Hippel–Lindau* (*VHL*) tumour suppressor gene and chromatin remodelling genes (*PBRM1*, *BAP1*, *and SETD2*).^5^ Mutations in *VHL* lead to stable activation of hypoxia‐inducible factor (HIF), which, in turn, upregulates vascular endothelial growth factor (VEGF), platelet‐derived growth factor beta (PDGF‐β) and transforming growth factor (TGF‐β).^6^ These represent the theoretical grounds of antiangiogenic therapy against VEGFR, PDGFR, and c‐KIT^7^ using the tyrosine kinase inhibitors (TKIs) sunitinib, sorafenib, and cabozantinib. Although TKI and immune checkpoint inhibition (ICI) combination therapy is the current treatment for metastatic RCC (mRCC) patients, sunitinib‐monotherapy is still an option for patients who are ineligible or unresponsive to ICI. Moreover, sunitinib improved the clinical outcome for patients in the International Metastatic RCC Database Consortium (IMDC) favourable‐risk group.^8^ Either way, most patients experience disease progression after initial response to treatment and tumour shrinkage.^9^

Mechanisms of adaptation and resistance to sunitinib encompass not only the tumour vasculature^9^, ^10^ but also epithelial tumour cells,^11^ such as drug‐lysosomal sequestration,^12^ via transcription factor EB (TFEB)^13^ and modulation of ABC transporter subfamily B member 1 (ABCB1) activity, thereby promoting drug efflux and autophagy inhibition.^14^ Intracellular pathways can be also reactivated via ‘bypass’ mechanisms that are independent on the sunitinib targets (reviewed in Yoda et al.^15^). Notably, the cross‐talk between VEGFR and other receptor tyrosine kinases or downstream signalling regulate epithelial‐to‐mesenchymal transition (EMT) and invasion, and cause TKI resistance in RCC.^16^, ^17^, ^18^

In addition to acquired genetic mutation, mounting observations have pointed to non‐genetic mechanisms being responsible for cell adaption and refractoriness to the treatment in various tumors.^19^, ^20^ This phenotypical state, known as ‘drug‐tolerant’, lies between drug sensitivity and resistance, and can potentially evolve (or speed up) into genetically stable acquired resistance.^21^, ^22^ The drug‐tolerant state is reversible, as the cells can resume their initial characteristics and drug sensitivity upon interruption of treatment (reviewed in Shen et al.^23^). These features offer novel therapeutic opportunities for second‐line treatments to target or even eradicate minimal residual disease (MRD).

While the specific resistance mechanisms of stable acquired resistance are increasingly being uncovered,^24^ it is not yet clear how a tumour cell population can survive during sunitinib treatment, emerge as a tolerant phenotype first, and potentially evolve into stably resistant cells afterwards. In this study, we modelled the acute and long‐term drug response in two human RCC cell lines using the IC_50_ dose of sunitinib continuously. We detected a cell population that maintained viability under treatment for 4 months. This phenomenon was reversible (summarized in Graphical Abstract, Figure S1). The characterisation of sunitinib‐tolerant cell models, compared to age‐matched treatment‐naive cells, provided valuable information on the diverse biological aspects of drug response and adaptation. Understanding the gradual refractory to treatment, on the molecular level, can advance not only the existing therapeutic approaches, but also improve the identification of the relapse state in mRCC. Ultimately, this approach can have significant implications in chronic control of the disease.

## 2 MATERIALS AND METHODS

### 2.1 Cell culture

Human renal cell carcinoma 786‐O and Caki‐2 cell lines were purchased from the American Type Culture Collection (LGC Standards, Teddington, UK). The 786‐O cells were cultivated in a Dulbecco's modified Eagle's medium (DMEM)/RPMI‐1640 mixture, and Caki‐2 cells in RPMI‐1640, both containing 10% heat‐inactivated fetal bovine serum (FBS) (Sigma Aldrich, St. Louis, MO, USA). Cellular growth conditions were set at 37°C and 5% CO_2_ humidification. Exponentially growing cells were used for all experiments. Sunitinib‐malate was purchased from Tocris Bioscience (Bristol, UK).

### 2.2 Induction of sunitinib tolerance in vitro

The IC_50_ (inhibitor concentration that kills 50% of cells) was determined following a sunitinib dose–response analysis (72 h). Next, the 786‐O and Caki‐2 cells were cultivated under the respective IC_50_ sunitinib for approximately 4 months to generate sunitinib‐tolerant cell lines. Sunitinib‐tolerant and age‐matched untreated, wild‐type cells were routinely sub‐cultivated, and their response towards sunitinib was compared every 2 weeks via a cell viability assay. During drug withdrawal, the cells were propagated in drug‐free media.

### 2.3 Cell viability

The cell lines were seeded into a 96‐well plate at densities of 1 × 10^3^ per well (786‐O) or 2 × 10^3^ per well (Caki‐2). Following cellular adherence overnight, the cells were exposed either to DMSO, increasing concentrations of sunitinib (0.1–10 μM), or to a single concentration of sunitinib (IC_50_) for 72 h. Next, the cells were incubated with WST‐1 solution according to the manufacturer instructions. Optical density was measured at 450 nm with a reference wavelength of 620 nm (microplate‐reader, Tecan Infinite Pro‐200, Switzerland).

### 2.4 Cell proliferation

The BrdU colorimetric kit (Merck KGaA‐Sigma‐Aldrich GmbH, Darmstadt, Germany) was used to evaluate new DNA synthesis following the incorporation of [3H]‐thymidine or 5′‐bromodeoxyuridine (BrdU) into DNA. In brief, after seeding (described in Section 2.3), the cells were treated with either DMSO or sunitinib for 24 and 72 h. At each time point, BrdU was added to the cells. The cells were processed for quantification of incorporated BrdU according to the manufacturer's instructions. BrdU incorporation was expressed using the optical density.

### 2.5 Caspase‐Glo® 3/7 assay

The cells were seeded and treated (see Sections 2.3 and 2.4) on a white‐walled plate (Corning, USA). Next, the cells were incubated at room temperature for 60 min with Caspase‐Glo® 3/7 Assay (Promega Corporation, WI, USA) to evaluate caspase activity. Luminescence signals were proportional to the amount of caspase activity.

### 2.6 Clonogenic assay

The cells were plated at a density of 100 cells per well (786‐O cells) or 700 cells per well (Caki‐2) in a six‐well plate. Following cellular adherence, the cells were exposed either to DMSO or to sunitinib for 24 and 72 h. The medium was then replaced with a standard growth medium, and the cells were allowed to recover for 9 days. The cells were fixed in 80% ethanol and stained with Coomassie‐Blue solution. Plating efficiency (PE) = (No. of colonies/number of cells seeded) × 100.

### 2.7 Cell migration and invasion

After serum starvation, 5 × 10^4^ of 786‐O and 1 × 10^5^ of Caki‐2 cell lines were seeded into a modified Boyden chamber (MilliporeSigma, Burlington, MA, USA) or Matrigel‐coated inserts (Corning Inc., NY, USA) with 8 μM pore size to evaluate cell migration and invasion, respectively. The lower compartment was filled with cell growth media supplied with 10% FBS. The cells were allowed to migrate under treatment with DMSO or sunitinib for 12 h. The cells on the outer side of the inserts were fixed in 75% ethanol and stained with crystal violet (Carl Roth GmbH, Germany). The cells were counted under a microscope at 10× magnification.

### 2.8 Flow cytometry analysis

Following treatment with DMSO or sunitinib for 48 h, the cells were harvested with trypsin–EDTA and washed in cold PBS. We fixed 1 × 10^6^/mL cells in 70% ice‐cold methanol. Propidium iodide staining was used to analyse the cell cycle distribution with flow cytometry. The results were evaluated with FlowJo software (BD Bioscience, CA, USA).

### 2.9 SDS‐PAGE and immunoblotting

Twenty micrograms of cell lysates were separated using SDS‐PAGE (Mini‐PROTEAN gel electrophoresis cell, Bio‐Rad Laboratories, CA, USA) and transferred onto a methanol‐activated PVDF membrane (Serva Electrophoresis GmbH, Heidelberg, Germany) with a semi‐dry transfer system (Bio‐Rad Laboratories, CA, USA). The membranes were incubated with primary antibodies against MET, phospho‐METY^1234/5^, AXL, phospho‐AXL^Y702^, phospho‐ERK1/2^T202/Y204^, phospho‐Akt^T308^, phosho‐Akt^S473^, phosphoS6^S235/6^, FAK, phosho‐FAK^Y397^, Vinculin, Cofilin, phospho‐CofilinS3, TESK1, phospho‐LIMK1/2^T508/T505^, beta‐actin and GAPDH (Cell Signalling Technology, NEB, Hitchin, UK), and BCL‐2 (Millipore, Sigma, Germany) with agitation overnight at 4°C. The secondary antibody: horseradish peroxidase (HRP)‐conjugated anti‐rabbit IgG (Cell Signalling Technology, Hitching, UK). Detection was performed via chemiluminescence (Serva Electrophoresis, Heidelberg, Germany).

### 2.10 Fluorescence staining

The cells were grown in a four‐chamber culture slide, fixed in 4% paraformaldehyde, washed in PBS, permeabilized with PBS‐0.1% Triton X‐100, and incubated with 1 mg/mL Bovine Serum Albumin (BSA) buffer. To visualize F‐actin, the cells were incubated with Phalloidin‐iFluor™ 488 Conjugate (AAT Bioquest®, Inc., Sunnyvale, USA) and mounted in Vectashield® Antifade Mounting Medium with DAPI (Vector Laboratories, Inc., Burlingame, USA).

### 2.11 Data evaluation and statistics

Dose–response and IC_50_ values were assessed with a four‐parameter nonlinear regression model. Analysis of variance (ANOVA) and Tukey's post‐hoc test were applied for a multiple comparison analysis using Graph Pad Prism®, and a *p*‐value of ≤0.05 was regarded as statistically significant. The data are presented as mean ± standard error (SE) of biological replicates, each performed in technical replicates. The graphs were generated with Graph Pad Prism® or in Excel. Image analysis software ImageJ (http://rsb.info.nih.gov/ij/index.html) was used to analyse the area of cells labelled with Phalloidin (Section 2.10), or to perform immunoblotting band densitometry. The cellular area was calculated by highlighting the cellular perimeter from microscopy images taken at 20×. Protein band densities from the immunoblotting analysis were determined as the optical density intensity (ODI) relative to the loading control (β‐actin or GAPDH). Protein abundance was expressed as the fold change between sunitinib‐tolerant and wild‐type cell lines. Vector‐based graphics editors, Inkscape 1.0.2–2 (https://inkscape.org) and BioRender (BioRender.com) were used to create schematic drawings and scientific illustrations.

## 3 RESULTS

### 3.1 Development of sunitinib‐tolerant human RCC cell lines

**FIGURE 1:**
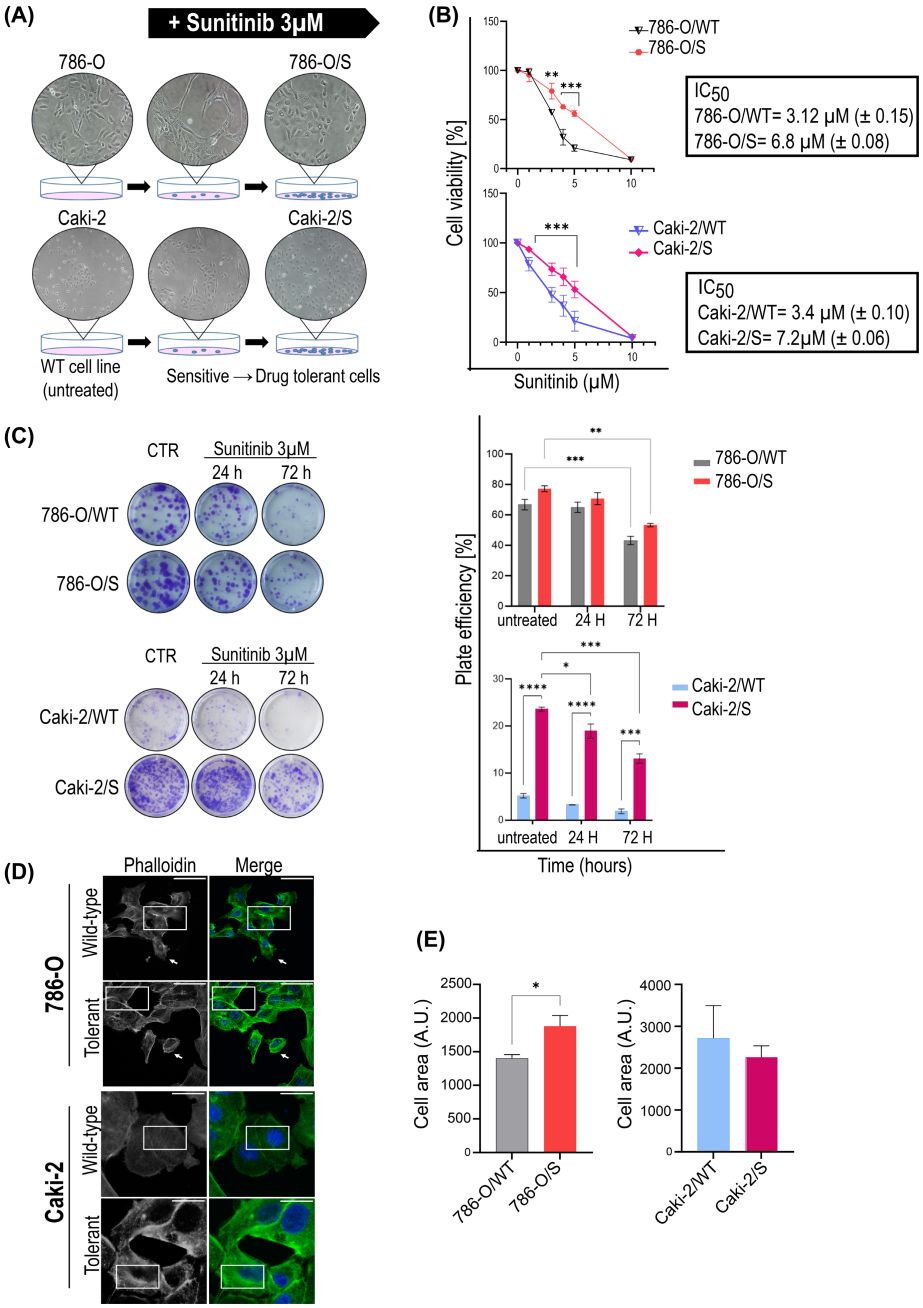
In vitro model of Sunitinib‐tolerance in RCC. The 786‐O/S and Caki‐2/S cells (A) were established after prolonged treatment (pictures from phase‐contrast microscopy) and showed increased IC_50_ compared to wild‐type cells (B). Clonogenicity of sunitinib‐tolerant and wild‐type cell lines (C). Average of percentage of plating efficiency (%) ± SE of three independent experiments (ANOVA test: ** *p*‐value <0.01 and ***p value <0.001 vs. DMSO, ***p‐value <0.001; **** *p*‐value <0.0001 versus wild‐type cells). Fluorescence labelling (D) with Phalloidin Alexa Fluor‐488 (green) was used to evaluate differences in Actin cytoskeleton (framed), and (E) in cell area (average ± SEM of eight independent fields, *p < 0.005). Confocal microscopy merged channels with DAPI (blue, nuclei). Scale bars: 20 μm.

The 786‐O and Caki‐2 cell lines were continuously exposed to the IC_50_ dose of sunitinib, corresponding to 3 μM (Figure S2). Within few days of treatment, a small fraction of the cell population survived, and was further cultivated under the selective pressure of sunitinib 3 μM. Over time, this population resumed proliferative ability under treatment and gave rise to expanded sunitinib‐tolerant 786‐O/S and Caki‐2/S cell lines (Figure 1A), which could be maintained and propagated in the presence of the drug for 4 months regularly. Our study included the untreated, wild‐type cell phenotype, that is 786‐O/WT and Caki‐2/WT, and the age‐matched 786‐O/S and Caki‐2/S. The IC_50_ of sunitinib in 786‐O/S and Caki‐2/S increased to 6.8 μM (± 0.08), and 7.2 μM (± 0.06), respectively (Figure 1B) by the end of the prolonged treatment, and their survival ability was further tested using a colony forming assay (Figure 1C). The 786‐O/S cells responded to 24 and 72 h of treatment with a plate efficiency of 72% (± 2.5) and 53% (± 0.9), respectively, as opposed to wild‐type 786‐O/WT cell lines (64 ± 2.7 and 43 ± 1.9). Remarkably, the Caki‐2/S cells gained a clonogenic ability that was fivefold greater than the Caki‐2/WT after 24 h of drug exposure (18% ±1.8 vs. 3.3% ± 1.4, singly), and this further increased by >6‐fold following the 72 h of treatment (13% ± 0.5 in the Caki‐2/S cells and 2% ± 1.9 in Caki‐2/WT cells). Taken together, the data indicate the emergence of cellular phenotypes that can survive continuous treatment with sunitinib. We observed that 786‐O/S and Caki‐2/S were not fully resistant, as sunitinib could still partially exert an inhibitory effect.

Morphological changes were also evaluated as traits of drug‐tolerance (Figure 1D,E). The 786‐O/S cells showed enrichment of stress fibres across the cell body, density of actin organization forming filopodia, and a significant increase in cell area compared to 786‐O/WT cells (Figure 1E, right panel). Long exposure to sunitinib did not affect the cell area of Caki‐2/S cells but the drug induced actin thickening on the transverse and dorsal cell arch (Figure 1E, left panel).

### 3.2 Evaluation of acute and prolonged sunitinib treatment on migration and invasion

**FIGURE 2:**
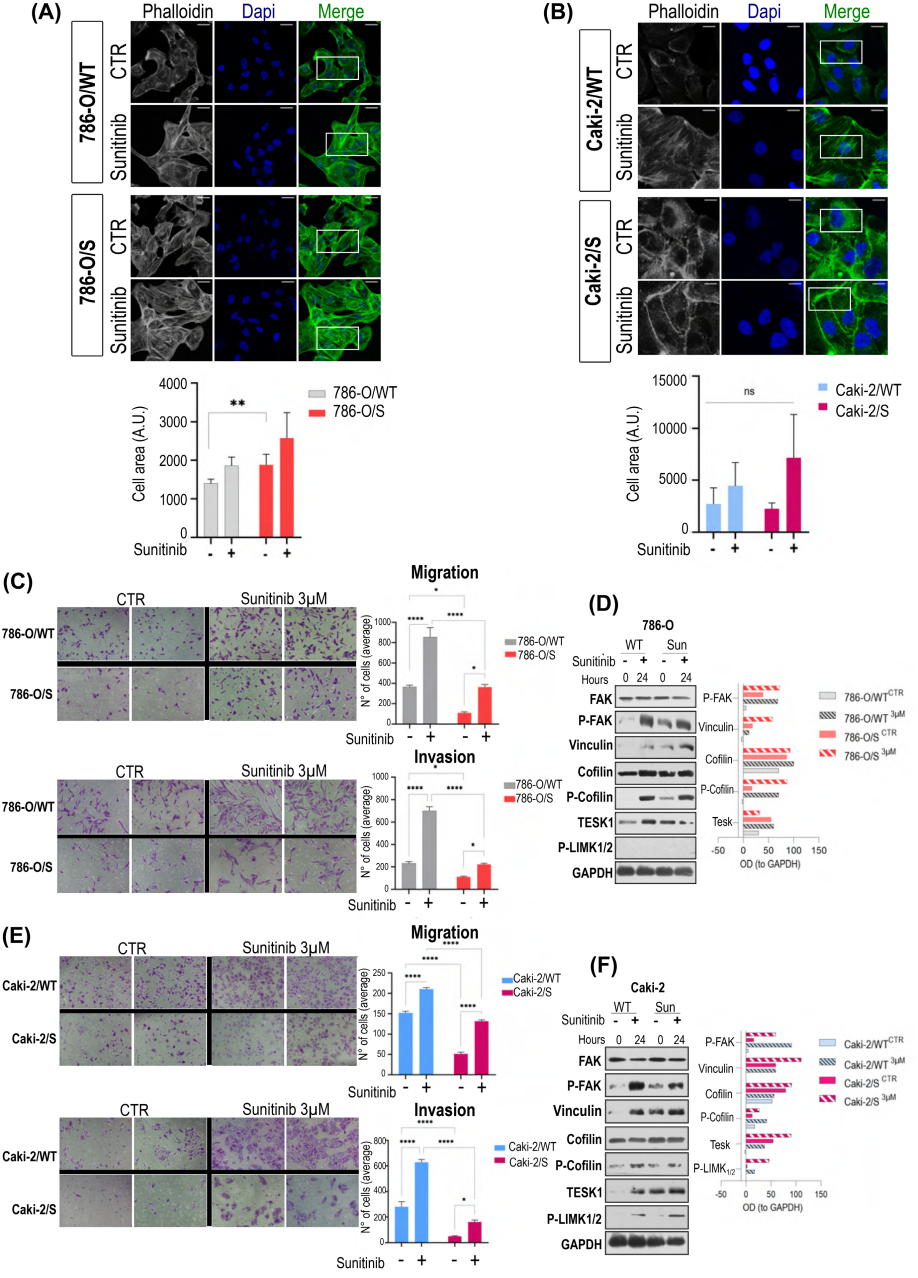
Sunitinib‐response was associated with actin remodelling, migration and invasion. Confocal microscopy (A, B) of actin filaments (Pallodin, green) in 786‐O/WT, 786‐O/S, Caki‐2/WT and Caki‐2/S treated with sunitinib, and relative quantification of cellular area (average ± SEM of six independent fields, *p < 0.005). Magnification at 60×. Cell migration and invasion (C–E) were assessed after DMSO or sunitinib treatment in 786‐O/WT, 786‐O/S, Caki‐2/WT and Caki‐2/S cells. (Cells counting under 10× magnification, *** *p*‐value <0.001 vs. DMSO; **p‐value <0.01 vs. WT). Immunoblotting of intracellular signalling in 786‐O/WT, 786‐O/S (D), Caki‐2/WT, and Caki‐2/S (F) left untreated (−) or with (+) 3 μM sunitinib (Sun) for 24 h. GAPDH expression was used as sample loading control and as a normalization value for densitometry analysis.

We reasoned that actin remodelling could be an acute response to sunitinib regardless of the cellular phenotype, as short‐term exposure to sunitinib (24 h) also provoked an increase in the density of stress fibres in the wild‐type cell lines (Figure 2A,B). We tested cell migration and invasion abilities that are important for metastasis and connected with actin dynamics (Figure 2C–E). The short‐term drug treatment promoted migration and invasion three‐fold regardless of the cell phenotype (Figure 2C,E). These observations were ascribed to activation of the molecular network regulating the actin cytoskeleton (Figure 2D–F). The active forms of focal adhesion kinase (pFAK^Y397^) and vinculin—important players for maturation of the focal adhesions (FAs)—were enhanced in both 786‐O/S and Caki‐2/S at the basal level (0 h of treatment) in contrast to the wild‐type counterparts. These data align with the thickening of cell fibres induced by prolonged exposure to sunitinib in 786‐O/S and Caki‐2/S (Figures 1D,E and 2A). Following 24 h incubation with 3 μM sunitinib, the level of those proteins was boosted in both wild‐type and tolerant cell lines (Figure 2D–F). Cofilin was analysed as a key regulator of actin depolymerization during membrane protrusion formation.^25^ Sunitinib treatment resulted in accumulation of phosphorylated cofilin, along with its regulators TESK1 and pLIMK1/2. Increased phosphorylation of cofilin during sunitinib treatment implies active actin polymerization and the occurrence of migration processes. Our data might suggest that cell migration and invasion are acute events possibly connected with securing tumour cell survival after short‐term treatment with sunitinib (24 h) but are not related to a sunitinib‐induced tolerant phenotype (long‐term drug exposure).

### 3.3 Analysis of proliferation and apoptosis in sunitinib‐induced tolerance in RCC cell lines

**FIGURE 3:**
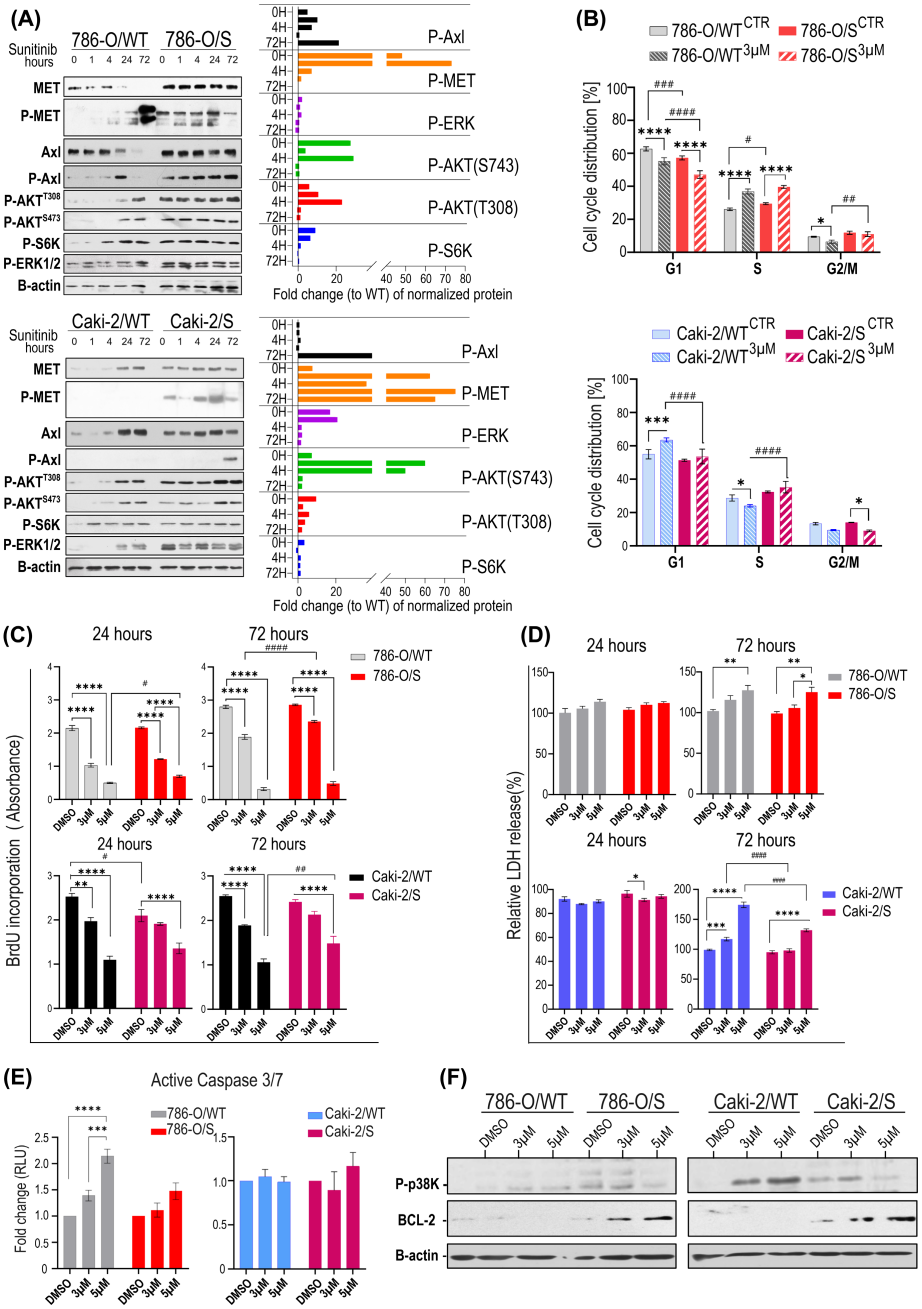
Effect of sunitinib‐tolerance on proliferation and cell death. Immunoblotting of MET and AXL signalling (A) in wild‐type and sunitinib‐tolerant cells left untreated (−) or with (+) sunitinib (Sun). β‐Actin: sample loading and normalization value for band densitometry and fold‐change calculation (to wild‐type). Flow cytometry analysis of cell cycle progression in 786‐O/WT and 786‐O/S and Caki‐2/WT and Caki‐2/S (B) after drug treatment. Cell proliferation (BrdU‐labelling, C), cytotoxicity (LDH‐released, D), and caspase 3/7 activity (E) were assessed in wild‐type and sunitinib‐tolerant cells at the indicated concentrations and times. Values: average ± SE of biological replicates. Statistics: two‐way ANOVA (*** *p*‐value <0.001 vs. DMSO; ^##^ *p*‐value <0.01 vs. wild‐type cells). Immunoblotting assay of BCL‐2 and p‐P38 proteins (F) in wild‐type cells and sunitinib‐tolerant cells at indicated concentrations; β‐Actin expression: sample loading control.

Within the complex network of bypass processes responsible for cell survival and proliferation in response to sunitinib, we expressly focused on mechanisms that could be exploited by current targeted therapies. This includes the multi‐receptor TKI targeting MET/AXL/VEGFR cabozantinib, which is currently recommended as second‐line therapy after failure of sunitinib as well as in combination with an immune checkpoint inhibitor as first‐ line therapy in patients with mRCC. We found that active P‐MET (phosphorylated MET^Y1234/5^) and P‐AXL (phosphorylated AXL^Y702^) receptors were endogenously (basal level) activated only in 786‐O/S and Caki‐2/S cell lines (Figure 3A), and that their phosphorylation was stabilized by further exposure to sunitinib. The level of total/phospho proteins suggested that the same portion of the two receptors was activated in both786‐O/S and Caki‐2/s cell lines. The receptors' activity reflected the modulation of the downstream signalling in sunitinib‐tolerant cells. The high expressions of phosphorylated ERK, AKT and‐S6K (mTOR marker) kinases were maintained through sunitinib incubation. Remarkably, we observed time‐dependent phosphorylation of the two receptor tyrosine kinases (RTKs) only in the 786‐O/WT under drug treatment. Interestingly, MET and AXL downstream signalling were induced following drug exposure in both wild‐type cell lines. These results might explain why cell colonies from the wild‐type cells were able to emerge after sunitinib treatment (24 and 72 h), although these were at reduced frequency. This indicates not only the importance of sunitinib‐induced MET and AXL signalling as an acute (non‐genetic) event, but also as a critical determinant for cellular viability and survival in a long‐term setting. Results from cell cycle profiling (Figure 3B) show that drug treatment significantly affected the S‐phase of the cell cycle in both 786‐O/S and sensitive 786‐O/WT cell lines (*p‐value ≤0.0001), whilst the 786‐O/WT line also responded with a drop in the cell population in the G2 phase (*p*‐value <0.01, Table 1). Thus, the ability of 786‐O/S cells to continue the cell cycle under treatment clearly depended on the acquisition of drug tolerance in the 786‐O cell model (two‐way ANOVA: *p*‐value 0.013). Sunitinib treatment induced a significant accumulation of Caki‐2/WT cells in the G1 phase of the cell cycle (* *p*‐value 0.001, relative to DMSO) but no significant change in Caki‐2/S, implying that the cytotoxic activity of sunitinib was exerted evenly across all phases of the cell cycle in the Caki‐2/S cell line.

**TABLE 1:** Cell cycle distribution in the wild type and sunitinib‐tolerant cell lines after treatment with DMSO and sunitinib.

	786‐O/WT ^CTR^	786‐O/WT ^3μM^	786‐O/S ^CTR^	786‐O/S ^3μM^
G1				
Mean (±SE)	64 (± 0.4)	56 (± 0.9)	58 (± 0.6)	48 (± 1.3)
*P_a_		<0.0001		<0.0001
^*#*^ *P*_b_			0.0002	<0.0001
S				
Mean (±SE)	27 (± 0.5)	37 (± 1.06)	30 (± 0.4)	41 (± 0.6)
*P_a_		<0.0001		<0.0001
^*#*^ *P*_b_			0.0238	n.s
G2/M				
Mean (±SE)	10 (± 0.1)	6 (± 0.5)	12 (± 0.4)	11 (± 0.8)
*P_a_		0.0478		0.0017
^*#*^ *P*_b_			n.s	n.s

	Caki‐2/WT ^CTR^	Caki‐2/WT ^3μM^	Caki2/S ^CTR^	Caki‐2/S ^3μM^
G1				
Mean (±SE)	57 (± 1.6)	65 (± 0.6)	53 (± 0.3)	55 (± 2.4)
*P_a_		0.0001		
^*#*^ *P*_b_			n.s	n.s
S				
Mean (±SE)	30 (± 1.12)	25 (± 0.5)	33 (± 0.4)	36 (± 2.1)
*P_a_		0.0358		n.s
^*#*^ *P*_b_			n.s	<0.0001
G2/M				
Mean (±SE)	14 (± 0.5)	10 (± 0.2)	14 (± 0.06)	9 (± 0.3)
*P_a_		n.s		0.022
^*#*^ *P*_b_			n.s	n.s

*Note*: Percentage of cell population (±SE). Statistical significance (multiple comparison anova): (a) *p‐value: treatment versus DMSO. (b) #*p*‐value (vs. wild type [WT] cells).

The concept of drug tolerance might imply the reversion to a sensitive state and re‐sensitizing of the tumour cells. Using two‐time points analysis, we compared proliferation and cell death between the wild type and sunitinib‐tolerant cells after exposure to 3 and 5 μM of sunitinib, which corresponded to ~IC_65_ of sunitinib in both wild type cell lines (no‐lethal dose), and to the ~ IC_40_ in the 786‐O/S and Caki‐2/S cell lines. The treatment with 3 μM sunitinib confirmed the gaining of drug tolerance in 786‐O/S, with an 18% reduction in proliferation compared to the 32% decrease in 786‐O/WT (two‐way ANOVA, ^(#)^ *p*‐value >0.0001). However, 786‐O/S could be re‐sensitized using 5 μM of sunitinib, like 786‐O/WT (Figure 3C). The ability of Caki‐2/S cells to proliferate remained unchanged under 3 μM sunitinib (in contrast to Caki‐2/WT, *p*‐value <0.001) until the cells were subjected to 5 μM (Figure 3C). Still, cell proliferation was less affected in Caki‐2/S cells (two‐way ANOVA, ^(#)^ *p*‐value 0.006 vs. Caki‐2/WT). Overall, these data indicate that sunitinib‐induced accumulation of the cell population in the S phase of the cell cycle (Figure 3B) is the result of cell growth arrest. The release of lactate dehydrogenase (LDH) in 786‐O/S and 786‐O/WT (Figure 3D upper panel) was similar when using 5 μM of the drug (72 h). In the Caki‐2/S cell line, LDH release was observed when only using 5 μM of sunitinib, and it was still significantly lower than that of Caki‐2/WT (^(#)^ *p*‐value <0.0001). The cellular response and partial restoration of drug sensitivity following 5 μM sunitinib were also confirmed by a dramatic change in cell morphology, including cell volume alteration or cellular fragmentation into membrane‐bound apoptotic bodies (Figure S3). The reduced drug sensitivity of 786‐O/S and Caki‐2/S was demonstrated by dose‐dependent upregulation of the anti‐apoptotic protein BCL‐2, and deactivation of apoptosis‐related p38 kinase (Figure 3E), in contrast to the wild‐type cells. Sunitinib dose‐dependent induction of Caspase 3/7 activity was found in the 786‐O/WT cell line, especially in response to 5 μM (*p*‐value <0.0001, to DMSO‐control). No significant apoptosis was reported in 786‐O/S under the same experimental conditions (Figure 3F), nor in the Caki‐2 cell line. Overall, the results confirm adaptation to the selective pressure of sunitinib in both 786‐O/S and Caki‐2/S. The boosting of proliferation and survival signalling might represent a reworking mechanism behind the therapy. The current evidence suggests a heterogeneous cell response to cellular stress induced by sunitinib.

### 3.4 Sunitinib‐tolerant phenotype was reversible

**FIGURE 4:**
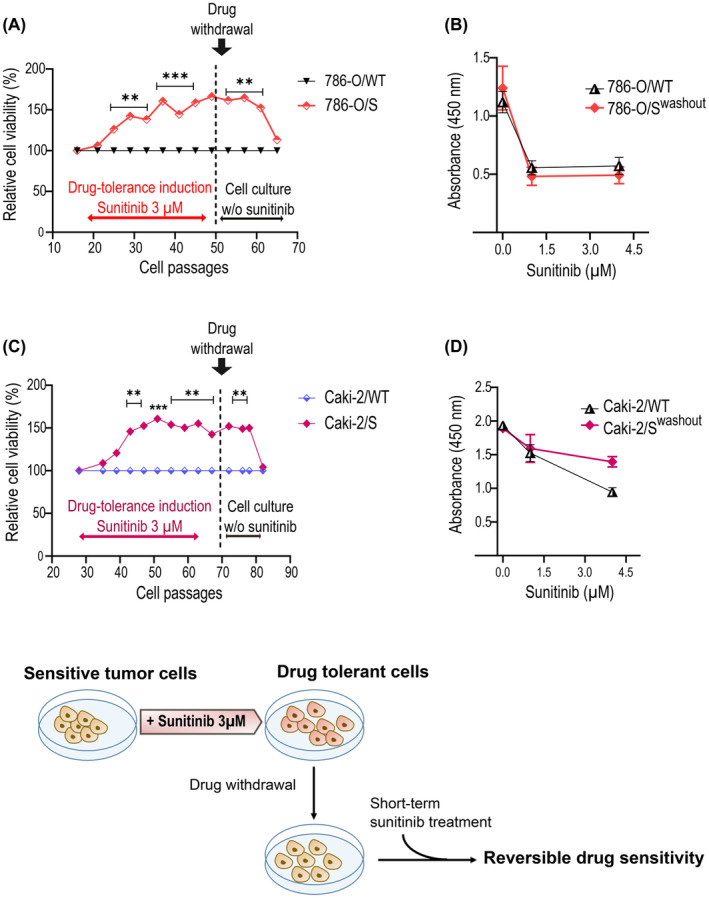
Reversibility of drug‐tolerant cell population. Cell survival rate of 786‐O/S (A) and Caki‐2/S (C) cell lines was routinely assessed throughout the drug‐tolerance induction (time of entering the sunitinib‐tolerant state). The drug‐tolerant rate was defined by normalizing the cell viability of 786‐O/S and Caki‐2/S to the wild‐type (used as reference samples). Cellular response to sunitinib was monitored during drug withdrawal for the indicated passages; a dose–response curve was created by the end of the drug washout (B, D). Values: mean ± SEM of biological replicates (*** *p*‐value <0.001; **p‐value <0.01 vs. WT).

The adaptation process leading to a drug‐tolerance state has been connected to non‐mutational molecular mechanisms and it might therefore be reversible. Consistent with this view, we monitored the response of 786‐O/S and Caki‐2/S to sunitinib after propagation in drug‐free media and compared this with the age‐matched wild‐type cell lines (Figure 4A,C). Both cell lines resumed drug sensitivity within 15 cellular passages under drug withdrawal. Interestingly, this change was rather sudden. The sunitinib‐tolerant cell phenotype showed temporal stability (about 10 passages), followed by a quick drop in cell survival. At this point, the 786‐O/S and Caki‐2/S cell lines could be successfully drug‐desensitised, similarly to the counterpart wild type (Figure 4B, D).

## 4 DISCUSSION

Failure of therapy with TKI inhibitors arises from different mechanisms. In mRCC, screening methods and therapeutic strategies to counteract unsuccessful treatment with sunitinib are generally focused on genetic mutation, like the loss‐of‐function of *G6PD*, *LRP1B*, *SETD2*, *TET2*, *SYNE1*, and *DCC* genes^26^ that generate fully resistant cell clones.^27^, ^28^ Non‐genetically driven phenotypes, the ‘residual’ drug‐tolerant cells, have not yet been described in mRCC during sunitinib treatment. Considering the relevance of the drug‐tolerant state for enduring drug pressure^29^ and for accelerating the transition to genetically stable resistance,^24^ we modelled a sunitinib‐tolerant state in vitro. Chronic exposure to the IC_50_ dose of sunitinib caused a switch to a drug‐tolerant state in 786‐O and Caki‐2 cell lines, which exhibited altered cellular morphology, DNA synthesis, susceptibility to apoptosis, dysregulated intracellular signalling, and reversible enhanced survival. As reported in lung cancer,^30^, ^31^ the 786‐O/S and Caki‐2/S cell lines straddled the sensitive and resistant states. In this regard, IC_50_ in 786‐O/S and Caki‐2/S cells increased by 2.3‐fold, which could be viewed as a small increase compared to what has previously been described in sunitinib‐resistant models.^32^ In contrast, our results demonstrate the enrichment of a specific cell population displaying drug adaptation and diverse biological properties. We found better clonogenicity in the sunitinib‐tolerant cells relative to the wild type cells, especially in the Caki‐2/S cells. Shan and colleagues showed that higher clonogenicity was a feature of sunitinib‐resistant cells, and it was biologically driven by lncRNA CCAT1,^32^ proving the engagement of drug‐induced epigenetic rearrangement for cell survival, proliferation the cell cycle, DNA replication/DNA repair, and metabolism.^20^, ^33^, ^34^ Remarkably, colonies of 786‐O/WT and Caki‐2/WT cell lines emerged following sunitinib treatment at low frequency. This might suggest that a sunitinib‐tolerant population could arise de novo, and possibly this was not related to stable genetic mutations. Notably, the sunitinib‐tolerant phenotype of the 786‐O/S and Caki‐2/S cells could exist only in the presence of sunitinib. The cells could be re‐sensitized following 15 cell passages of drug washout, which implies that non‐genetic reprogramming induced by cellular stress might enable the tumour cells to transition from a sensitive to a tolerant phenotypic state. Sharma et al.^35^ reported on chromatin alteration and functional cell heterogeneity as strategies to transitorily acquire TKI‐tolerant cells, as opposed to stable genetic alteration. Screening for epigenetic alterations during the development of tolerance to sunitinib in RCC cell models was beyond the scope of our study, and further investigations are needed especially, with regard to novel biomarker identification for prediction and therapy response.

The phenotype of 786‐O/S and Caki‐2/S cells relies on receptor tyrosine kinases c‐MET and AXL signalling, which might influence the cellular drug response in terms of cell proliferation and cell death. The sunitinib‐tolerant phenotype showed reduced inhibition of DNA synthesis under treatment, compared to the wild‐type confirming the activity of sunitinib on cell proliferation.^36^, ^37^ In various tumour entities, aberrant activation of MET and AXL has been described in terms of resistance to RAS–RAF–MEK, mammalian target of rapamycin (mTOR), VEGFR therapies^16^, ^38^, ^39^ including sunitinib and sorafenib,^16^, ^40^ and, recently, immune evasion.^41^ The activity of c‐MET‐downstream kinases, ERK ½, and AKT/S6K might also be related to enhanced cell survival and proliferation in 786‐O/S and Caki‐2/S cells, as previous studies have reported,^42^ also concerning pathogenesis and sunitinib resistance in RCC.^43^, ^44^, ^45^ A distinct ability to overcome cell death is a further trait of sunitinib tolerance. We observed a sunitinib dose‐dependent increase in the anti‐apoptotic protein BCL‐2,^46^ while the pro‐apoptotic kinase p38^47^ was stepwise downregulated. We found a heterogeneous apoptotic response (caspases 3/7) between 786‐O and Caki‐2 cell lines. More investigation should be performed in this respect, considering that the engagement of caspases might depend on variation in the time required for cell death. No dramatic changes were found in cell cycle profiling, which could imply that both sunitinib‐tolerant cell lines resumed proliferation, as reported in a previous study.^48^ Overall, we were able to show that tolerant cells gained the ability to expand indefinitely during constant sunitinib exposure. Nevertheless, we acknowledge two important points. First, a significant reduction in proliferation was observed in 786‐O/S and Caki‐2/S cells in response to increasing concentrations of sunitinib, confirming once again that our cell models are in a transitional state between sensitivity and resistance. From a clinical perspective, this outcome might support retreatment after a ‘drug holiday’, or a dose escalation. Second, sunitinib triggered a time‐dependent expression of c‐MET and AXL pathways in treatment‐naïve cell lines. Therefore, this signalling could not only protect cells from early drug toxicity but also orchestrate further selection of cell clones that endure sunitinib‐induced stress. Upregulation of c‐MET and AXL in a sunitinib‐resistant state^16^ has provided a rationale for second‐line therapy with the c‐MET/AXL/VEGFR inhibitor cabozantinib. However, we have recently reported that a c‐MET and AXL‐driven status did not improve cell responsiveness to cabozantinib, and intracellular signalling for cell proliferation and survival, including Src‐FAK activation, was triggered.^49^ In this study, FAK was also activated after acute and prolonged treatment with sunitinib, suggesting similar redundant strategies of the cellular response to targeted therapy with TKIs.

Alteration in actin cytoskeleton dynamics and interaction with the extracellular matrix (ECM) have been associated with drug response and resistance.^50^, ^51^, ^52^ The cell size, stress fibre organization, and activity of actin‐regulators were altered in sunitinib‐tolerant cells when compared with parental cells. Nonetheless, the actin filament patterns showed dynamic regulation even during short‐term treatment. A remarkable example is the regulation of focal adhesion kinase (FAK) and vinculin, which mediate adhesion and motility signals.^53^ The protein FAK is crucial for cell survival, lamellipodia, organization of cytoskeleton components, and initiation of metastasis in many solid tumors.^54^, ^55^ In sunitinib‐tolerant cells, the high basal level of P‐FAK was further increased after drug exposure, whereas the protein was boosted sevenfold in wild‐type cells after treatment. These observations could explain the augmented migration and invasion ability in all analysed cells, as these processes rely on the dynamics of F‐actin polymerization (initiating lamellipodia and filopodia), and the formation and stabilization of focal adhesions to the ECM.^56^ On the one hand, our data show that the capability of RCC cells to evade treatment might represent an early phase of the drug response to sunitinib through the activation of focal adhesion molecules. On the other hand, additional applications of the drug to sunitinib‐tolerant RCC cells might stimulate further metastatic potential. We have highlighted the importance of addressing the drug‐tolerant sunitinib phenotype as it has the closest resemblance to MRD in clinics.^31^ A sunitinib‐tolerant population could arise by adopting characteristics that could prevent the eradication of cancer cells by lethal exposure to sunitinib.

Although our in vitro study evaluated different aspects of the sunitinib‐induced tolerant phenotype, the findings have some limitations; for example, the rapid acquisition of sunitinib refractory relays on drug efflux via MDR1, ABCG2 transporters,^57^, ^58^ and lysosomal sequestration.^12^, ^14^ Further information must be provided via immunoblotting or gene expression assay. The level of matrix metallopeptidase 9 (MMP‐9) should be tested to better emphasise ECM remodelling as the cause of the dramatic increase in invasion during the acute phase of treatment. However, in this study we aimed at the potential clinical and therapeutic implications of signalling pathways alteration to counteract drug tolerance on sunitinib and prevent tumour recurrence or therapy failure.

Our findings suggest that the evasion of cell death and actin remodelling are distinct molecular features that could be regarded as therapeutic vulnerability to eradicate sunitinib‐tolerant tumour cells. Targeting BCL‐xL‐/BCL‐2 could sensitize tumour‐resistant cells to EGFR inhibitors,^21^ or result in the inhibition of actin remodelling molecules counteracting adaptive resistance to BRAF inhibitors in melanoma.^59^ Our findings highlight a need for further investigation on the epigenetic state as a possible strategy to fight TKI responsiveness in RCC.

## AUTHOR CONTRIBUTIONS

**Angela Zaccagnino:** Data curation (equal); formal analysis (lead); methodology (equal); writing – original draft (lead). **Bozhena Vynnytska‐Myronovska:** Data curation (equal); methodology (equal); writing – review and editing (supporting). **Michael Stöckle:** Conceptualization (supporting); funding acquisition (supporting). **Kerstin Junker:** Conceptualization (lead); funding acquisition (lead); investigation (equal); project administration (lead); supervision (lead); writing – review and editing (equal).

## FUNDING INFORMATION

This research was funded by Alexander von Humboldt, Stiftung/Foundation, and awarded to Bozhena Vynnytska‐Myronovska.

## CONFLICT OF INTEREST STATEMENT

The authors confirm that there are no conflicts of interest or personal relationships that could have been perceived as potentially influencing the research presented in this paper.

## Supporting information


FigureS1.

FigureS2.

FigureS3.

## Data Availability

The data that supports the findings of this study are available in the supplementary material of this article.
