# Supplementary material for: Molecular and functional characterization of reversible‐sunitinib‐tolerance state in human renal cell carcinoma
Source: J Cell Mol Med. 2024 May 2;28(9):e18329. doi: 10.1111/jcmm.18329 (PMC11063727; doi:10.1111/jcmm.18329)

Graphical Abstract

Sunitinib treatment induces phenotypic transition of treatment-naive cells to drug-tolerant cells.
The drug-tolerant state is a reversible phenotype, as the cells can be re-sensitized after a drug washout phase.
The emergence of drug tolerance requires MET, AXL signalling and involved distinct cytoskeleton organization mediated by Focal adhesion kinases.

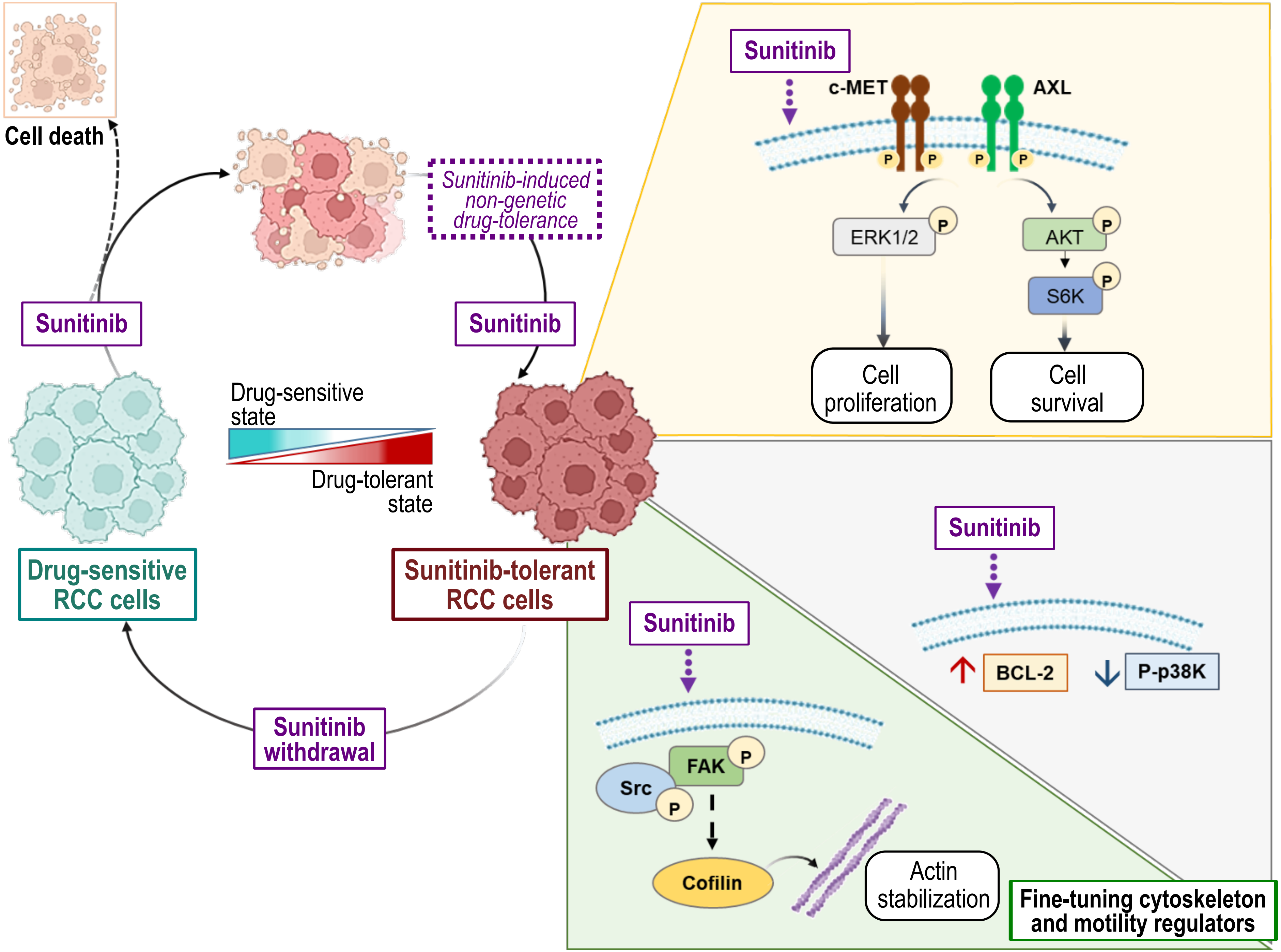

Supplement: Supplementary file 1 — FigureS1. [file JCMM-28-e18329-s002.pdf]
