# Supplementary figures and images for: Molecular and functional characterization of reversible‐sunitinib‐tolerance state in human renal cell carcinoma
Source: J Cell Mol Med. 2024 May 2;28(9):e18329. doi: 10.1111/jcmm.18329 (PMC11063727; doi:10.1111/jcmm.18329)

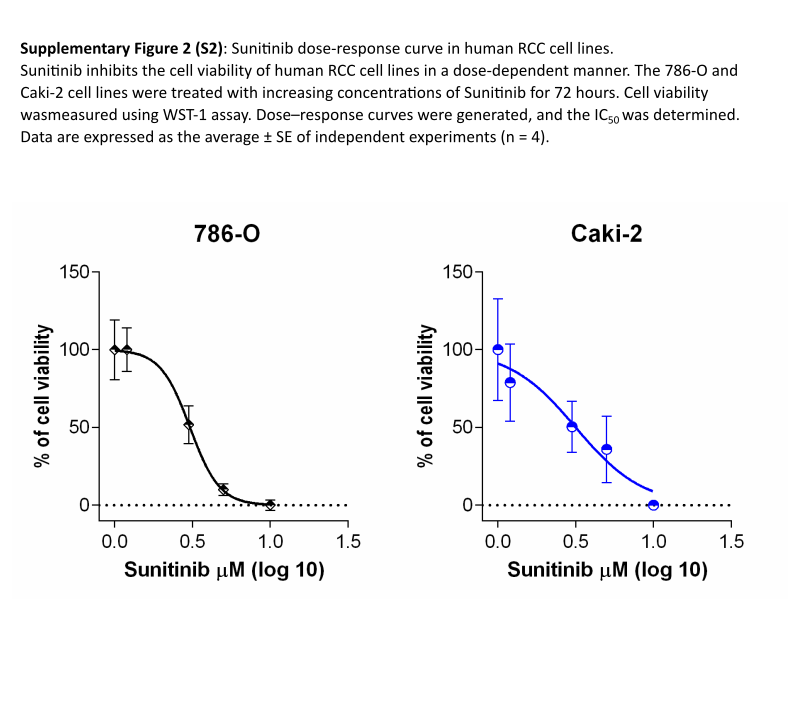

Supplement: Supplementary file 2 — FigureS2. [file JCMM-28-e18329-s003.tif]

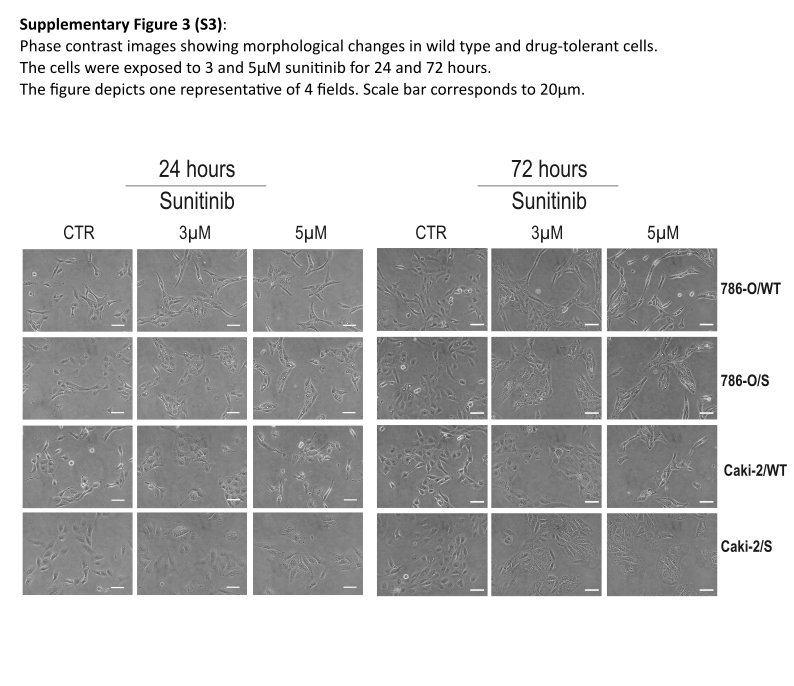

Supplement: Supplementary file 3 — FigureS3. [file JCMM-28-e18329-s001.tif]
